# Blood consumption in total arterial coronary artery bypass grafting

**DOI:** 10.1186/s13019-020-1053-1

**Published:** 2020-01-17

**Authors:** Raphael Sven Werner, Christoph Lipps, Stefan Waldhans, Andreas Künzli

**Affiliations:** 1 Department of Thoracic Surgery, University Hospital Zurich, Rämistrasse 100, 8091 Zurich, Switzerland; 2 Department of Cardiovascular Surgery, Herzzentrum Bodensee, Kreuzlingen, Switzerland

**Keywords:** Coronary artery bypass, Arterial bypass grafting, Internal mammary artery, Blood transfusion

## Abstract

**Background:**

Accumulating evidence consistently demonstrates that blood transfusion in cardiac surgery is related to decreased short- and long-term survival. We aimed to evaluate periprocedural blood loss and transfusion rates in elective, isolated total arterial coronary artery bypass grafting (CABG) using exclusively skeletonized bilateral internal mammary arteries (IMAs).

**Methods:**

We identified 1011 consecutive patients with coronary artery disease who underwent CABG between 1/2007 and 12/2014. Of them, 595 patients who presented preoperative hemoglobin levels >9md/dl and underwent elective, isolated CABG for multi-vessel coronary artery disease were included in the study population. 419 patients (70.4%) received total arterial CABG using skeletonized bilateral IMAs, in 176 patients (29.6%) mixed CABG (single IMA & saphenous vein) was performed. Propensity score adjustment using 16 variables was applied to control for treatment effect.

**Results:**

In patients undergoing total arterial CABG, heterologous blood transfusion could be avoided in 87.8% of all cases. Propensity score adjusted results showed a significantly lower incidence of erythrocyte concentrate transfusion in patients undergoing total arterial CABG compared to mixed CABG (odds ratio 2.74, 95% confidence interval 1.38–5.43, *P* = 0.004). There were no statistically significant differences in the rates of thrombocyte concentrate (*P* = 0.39) and fresh frozen plasma transfusions (*P* = 0.07).

**Conclusions:**

In this study, patients who underwent elective, isolated total arterial CABG using exclusively skeletonized bilateral IMAs showed reduced transfusion rates of erythrocyte concentrates compared to mixed CABG using a combination of single IMA and saphenous vein grafts. No evidence for a higher incidence of complications was found with a total arterial approach.

## Introduction

Blood loss is still one of the most frequent and feared complications in cardiac surgery leading to the consumption of a significant proportion of all blood products worldwide [1]. While blood transfusions are crucial in salvage procedures, they come with an increased risk of adverse effects such as the formation of alloantibodies and hemolytic transfusion reactions, allergic transfusion reactions, transfusion-associated circulatory overload, transfusion-related acute lung injury or the transmission of infections and transfusion-related immune modulation [2–5]. Accumulating evidence therefore demonstrates that a liberal strategy of red-cell transfusion in cardiac surgery shows no apparent benefits but is rather related to decreased short- and long-term survival [6–8].

Even the transfusion of a single blood unit has been shown to increase mortality and the length of hospital stay after CABG [9]. Results from both the Transfusion Requirements After Cardiac Surgery (TRACS) trial and the TRICS III Trial showed no inferiority of a restrictive transfusion strategy in cardiac surgery [10, 11] with respect to a composite outcome of death, stroke, myocardial infarction and acute renal failure. Furthermore, the Transfusion Indication Threshold Reduction (TITRe2) trial found no increase in the primary outcome of serious infections and/or ischemic events with a restrictive strategy [12].

According to numerous retrospective studies, total arterial CABG using bilateral internal mammary arteries (BIMA) is associated with improved long-term graft patency, long-term survival and reduced risk for cardiac events and cardiac death compared to mixed CABG using single internal mammary artery (SIMA) and saphenous veins (SV) [13–15]. While the first randomized trial on CABG survival at 10 years between BIMA and SIMA, the Arterial Revascularization Trial (ART), still awaits its outcome data, the analysis of clinical and safety outcomes after 5 years shows no significant differences in the rates of death, stroke or myocardial infarction between the bilateral- and single-arterial graft groups [16]. However, until now a total arterial approach is still chosen in less than 10% of all CABG procedures performed in Europe [17]. In consideration of the high SV graft failure rate of up to 30% between 12 and 18 months (as reported in the PREVENT IV trial [18]), we perform total arterial CABG with IMAs as a preferential treatment in all patients assigned for coronary revascularization in our center.

While the goal of a bloodless intervention has brought up numerous blood-saving procedures and systems, the ideal surgical technique has yet not been addressed appropriately. Since there are currently no prospective trials examining blood product utilization in patients undergoing total arterial revascularization, the objective of this study was to evaluate transfusion rates in total arterial CABG using exclusively skeletonized BIMA compared to mixed CABG using a composition of SIMA and SV conduits.

## Patients and methods

After obtaining approval by the local research ethics committee (Project-ID: 2017–00929), we reviewed a single-center database containing a total of 1011 adult CABG procedures performed between January 2007 and December 2014 at the Cardiac Center Bodensee, Switzerland. Data were collected retrospectively.

### Patient selection

**Fig. 1:**
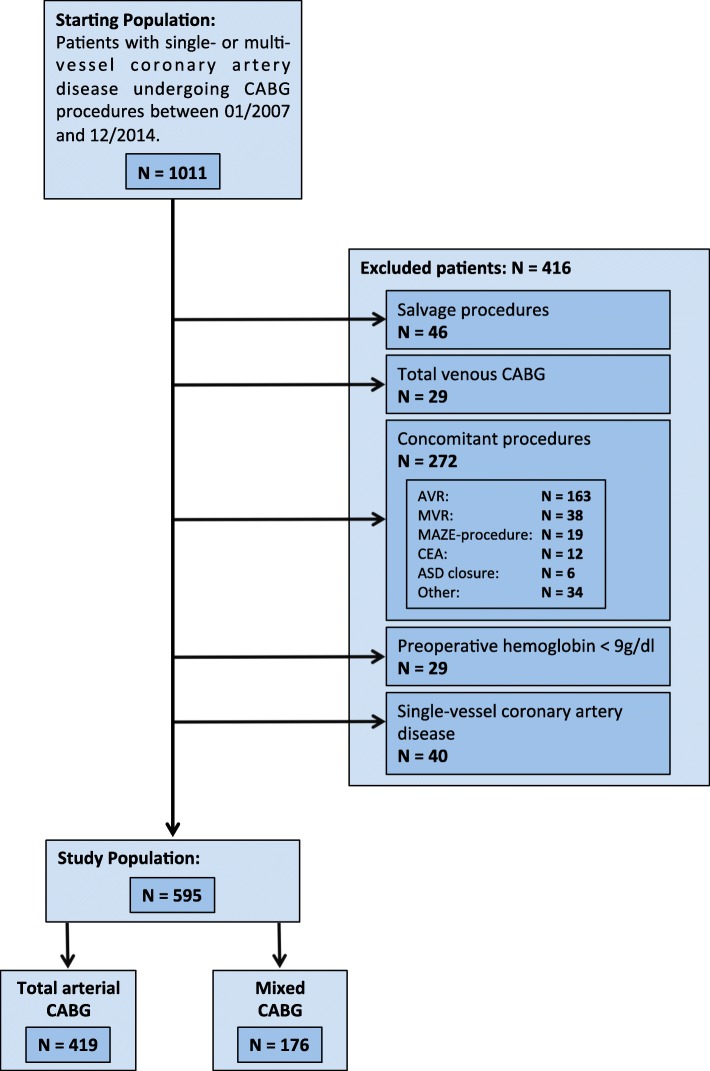
Flow diagram showing exclusion criteria and study group breakdown. AVR: aortic valve replacement, ASD: atrial septal defect, CEA: carotid endarterectomy, MVR: mitral valve reconstruction

From the starting population of 1011 patients we excluded all patients who were not amenable to an all-arterial approach, such as patients undergoing salvage procedures (*N* = 46) or total venous CABG using exclusively SV grafts (*N* = 29). We excluded all patients who were prone to experience increased blood loss due to concomitant procedures (*N* = 272) or who were prone to receive blood products due to low preoperative hemoglobin levels (*N* = 29). We excluded patients with single-vessel coronary artery disease (*N* = 40) since these patients were commonly only grafted with a single conduit. The study population included only patients with multi-vessel coronary artery disease who underwent elective, isolated CABG procedures. Full exclusion criteria and study group breakdown are shown in Fig. 1.

Within our study population, patients were attributed to two groups. The group undergoing total arterial CABG includes patients who were revascularized using exclusively BIMA conduits (*N* = 419) and the mixed CABG group includes patients who received a composition of SIMA and SV conduits (*N* = 176).

### Study endpoints

Primary endpoints were defined as periprocedural transfusion rates of packed red blood cells (erythrocyte concentrates, EC), thrombocyte concentrates (TC) and fresh frozen plasma (FFP) between the two groups during primary hospitalization. Periprocedural transfusion was defined as the transfusion of any of the above-mentioned blood products between the beginning of the cardiac surgery and the discharge from hospital. As secondary endpoints we assessed early outcomes, including 30-day all-cause mortality, stroke, perioperative myocardial infarction, deep sternal wound infection requiring surgical revision and surgical revision of hemorrhage.

Baseline data of preoperative anticoagulation in consideration of plasma half-life were listed in five groups: Acetylsalicylic acid (discontinuation time: 7d), ADP receptor inhibitors (discontinuation time: 7d (Prasugrel), 5d (Clopidogrel and Ticagrelor)), Vitamin K antagonists (discontinuation time: 7d (Phenprocoumon), 3d (Acenocoumarol)), Factor Xa inhibitors (discontinuation time: 2-7d (depending on renal and hepatic function) and GPIIb/IIIa inhibitors (discontinuation time: 6 h).

### Surgical technique

Median sternotomy was performed in all patients. All IMAs were skeletonized from the subclavian artery to the bifurcation and either harvested as free conduits for composite grafting or used as in situ grafts. Double clipping was performed on the side branches and topical papaverine and intraluminal heparinized solution of Ringer’s Lactate was applied for graft viability and spasm prophylaxis. SV grafts were harvested by an open method using double clipping. All procedures were performed on a conventional cardiopulmonary bypass using single aortic cross-clamping with cold blood cardioplegia. Moderate systemic hypothermia was induced. The grafting strategy and the use of total arterial or mixed CABG primarily depended on IMA graft quality and length. Several different graft configurations were used depending on the anatomy, configuration of coronary lesions, heart volume and total graft length needed.

For blood saving we used retrograde autologous priming on the cardiopulmonary bypass, reducing the standard priming volume to approximately 400 ml. Furthermore, the Cell Saver autotransfusion system was applied in redo-cases and in patients under dual antiplatelet therapy. Intraoperatively, all patients received tranexamic acid in a continuous scheme. Monofilament suture lines were used for all cannulation sites and all anastomoses. In all patients, CABG and the control of hemostasis was performed by the attending surgeon (A.K.) or under his supervision. No sealant at all was used for hemostasis and clipping was preferred for bleeding control whenever possible.

As recommended by the current clinical practice guidelines for blood transfusion, we followed a restrictive transfusion strategy perioperatively using a hemoglobin threshold of 7 g/dl in patients with a hemodynamically stable anemia. After cardiopulmonary bypass, indication for platelet transfusion was driven by clinical criteria such as substantial microvascular bleeding and/or excessive postoperative anemia in combination with a low platelet count < 50*10^9^/l and/or antiplatelet therapy. Indication for FFP transfusion was given for correction of substantial microvascular bleeding, especially in the presence of coagulation factor deficiencies. The transfusion strategy was identical in all patients and did not depend on grafting strategy.

### Statistical analysis

Continuous variables are reported as mean ± standard deviation and were compared using the unpaired t-test for normal distributions and Mann-Whitney U test for non-normal distributions. Categorical variables are expressed as frequencies and percentages and were compared using Chi^2^-Pearson-test.

Binary logistic regression was used to identify odds ratios of crude comparisons. To minimize the effect of potential confounding due to baseline dissimilarities or treatment selection, a propensity score adjustment was performed. Using binary logistic regression, a propensity score was generated for each patient with the treatment group as the dependent variable and sixteen independent variables (covariates). A logistic model was applied for the estimation of the propensity score. Covariates that were assumed to influence perioperative blood loss were chosen without previous knowledge of the clinical outcomes (outcome blinded). Covariates included the baseline clinical variables age, sex, era, arterial hypertension, diabetes, dyslipidemia, obesity, preoperative left ventricular ejection fraction (LVEF), perioperative anticoagulation (in five groups), number of distal anastomoses, preoperative hemoglobin levels, aortic cross-clamp time. Propensity adjustment was performed by logistic regression with the incidence of transfusion as the dependent variable and the treatment group and the propensity score on a logit scale as the independent variables. The validity of the logistic regression was assessed using the Hosmer-Lemeshow test. All reported *p* values are two-sided and a value of *p* < 0.05 was considered statistically significant. Statistical analysis was performed in IBM SPSS Statistics Version 22 (SPSS Inc., Chicago, IL).

## Results

### Baseline profiles

The baseline clinical profile of the total arterial and mixed CABG groups as well as the entire cohort is presented in Table 1. The two groups showed similar distribution of sex, age, hypertension, obesity, dyslipidemia, crossclamp time and anticoagulation scheme. Preoperative hemoglobin levels were not significantly different between the two groups. Patients undergoing mixed CABG showed lower preoperative LVEF and were more likely to be diabetic. Of all patients, 91.9% (91.4% in the total arterial and 93.2% in the mixed group) were under active anticoagulation or antiaggregation during cardiac surgery. The anticoagulation and antiaggregation regimen mainly comprised acetylsalicylic acid and ADP receptor inhibitors (83.7 and 20.3% of all patients, respectively).

**Table 1:** Baseline clinical profile of the entire cohort

Variable	Entire cohort *N* = 595	Total arterial CABG *N* = 419 (70.4%)	Mixed CABG *N* = 176 (29.6%)	*p* value
Male	501 (84.2%)	360 (85.9%)	141 (80.1%)	0.08
Age (y)	66.1 ± 9.5	66.0 ± 9.5	66.5 ± 9.5	0.55
< 60	139 (23.4%)	100 (23.9%)	39 (22.2%)	–
60–69	230 (38.7%)	167 (39.9%)	63 (35.8%)	–
70–79	186 (31.3%)	124 (29.6%)	62 (35.2%)	–
≥ 80	40 (6.7%)	28 (6.7%)	12 (6.8%)	–
Diabetes	162 (27.2%)	105 (25.1%)	57 (32.4%)	0.016
Hypertension	427 (71.8%)	299 (71.4%)	128 (72.7%)	0.07
Obesity (BMI > 30 kg/cm2)	197 (33.1%)	139 (33.2%)	58 (32.9%)	0.58
Dyslipidemia	385 (64.7%)	275 (65.6%)	110 (62.5%)	0.67
Preoperative hemoglobin	13.2 ± 1.8	13.3 ± 1.7	13.0 ± 1.8	0.07
Preoperative LVEF	52.0 ± 13.1	54.5 ± 11.8	45.7 ± 14.2	< 0.001
Anticoagulation and antiaggregation				
No anticoagulation or antiaggregation	48 (8.1%)	36 (8.6%)	12 (6.8%)	0.60
Acetylsalicylic acid	498 (83.7%)	359 (85.7%)	139 (79.0%)	0.61
ADP receptor inhibitors	121 (20.3%)	87 (20.8%)	34 (19.3%)	0.96
Vitamin K antagonists	9 (1.5%)	5 (1.2%)	4 (2.3%)	0.28
Factor Xa inhibitors	12 (2.0%)	6 (1.4%)	6 (3.4%)	0.09
GPIIb/IIIa inhibitors	26 (4.4%)	19 (4.5%)	7 (4.0%)	0.87
No. of distal anastomoses	3.4 ± 1.0	3.1 ± 0.9	3.9 ± 0.9	< 0.001
2	127 (21.3%)	117 (27.9%)	10 (5.7%)	–
3	221 (37.1%)	170 (40.6%)	51 (29.0%)	–
4	168 (28.2%)	96 (22.9%)	72 (40.9%)	–
5	70 (11.8%)	34 (8.1%)	36 (20.5%)	–
6	9 (1.5%)	2 (0.5%)	7 (4.0%)	–
Crossclamp time (min)	78.2 ± 27.8	79.0 ± 28.9	76.2 ± 25.2	0.25
Composite grafting	208 (35.0%)	200 (47.7%)	8 (4.5%)	< 0.001

*LVEF* left ventricular ejection fraction

### Surgical details

The mean numbers of distal anastomoses were 3.1 ± 0.9 in the total arterial CABG group and 3.9 ± 0.9 in the mixed CABG group (*p* < 0.001). In patients receiving a SV graft, sequential grafting was performed in 68.6% of all patients, with 2 distal anastomoses in 41.8%, 3 distal anastomoses in 20.6% and 4 distal anastomoses in 6.2% of all patients. In 31.4% of the patients receiving a SV graft, only one distal anastomosis was made. Composite grafting was performed significantly more often in patients undergoing total arterial CABG with a total of 200 cases (47.7%), compared to mixed CABG where composite grafting was performed in 8 cases (4.5%, *p* < 0.001). In total arterial CABG, one IMA was used as an in-situ graft in 124 patients (29.6%) and both IMAs were used as in-situ grafts in 286 patients (68.3%).

Similar aortic crossclamp times were found in the total arterial and the mixed CABG group (79.0 ± 28.9 min and 76.2 ± 25.2 min, respectively, *P* = 0.25). However, no significant difference in the aortic crossclamp time was found between patients where erythrocyte concentrates were transfused and patients who did not require EC transfusion (80.2 + 25.4 min and 77.9 + 28.2 min, respectively, *p* = 0.45).

### Clinical outcomes

Crude comparisons of early outcomes and blood loss are presented in Table 2. A significant reduction in the need for surgical revision of hemorrhage (0.5% vs. 2.8%, *p* = 0.015) was found in the total arterial CABG group compared to the mixed CABG group.

**Table 2:** Early outcomes and variables of perioperative blood loss of the unadjusted cohort

Variable	Entire cohort *N* = 595	Total arterial CABG *N* = 419 (70.4%)	Mixed CABG *N* = 176 (29.6%)	*p* value
Early postoperative outcomes				
All-cause 30-day mortality	3 (0.5%)	1 (0.2%)	2 (1.1%)	0.16
Postoperative MI	1 (0.2%)	1 (0.2%)	0 (0.0%)	0.52
Postoperative stroke	8 (1.3%)	6 (1.4%)	2 (1.1%)	0.78
Sternal infection	3 (0.5%)	2 (0.5%)	1 (0.6%)	0.89
Return for bleeding	7 (1.2%)	2 (0.5%)	5 (2.8%)	0.015
Incidence of transfusions				
EC-transfusion	104 (17.5%)	51 (12.2%)	53 (30.1%)	< 0.001
TC-transfusion	15 (2.5%)	6 (1.4%)	9 (5.1%)	0.006
FFP-transfusion	23 (3.9%)	10 (2.4%)	13 (7.4%)	0.003
Transfused units				
EC-units (95th percentile)	3	2	5	< 0.001
TC-units (97th percentile)	0	0	2	0.007
FFP-units (97th percentile)	2	0	3	0.003

*EC* erythrocyte concentrate, *FFP* fresh frozen plasma, *MI* myocardial infarction, *TC* thrombocyte concentrate

**Fig. 2:**
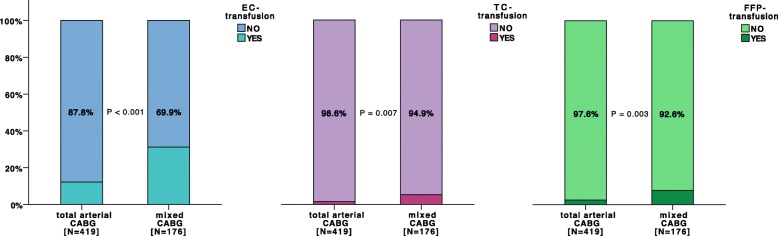
Histiogram depicting periprocedural transfusion rates of erythrocyte concentrates (EC), thrombocyte concentrates (TC) and fresh frozen plasma (FFP) during primary hospitalization. The data represent the unadjusted study population (*N* = 595, total arterial CABG: *N* = 419, mixed CABG: *N* = 176)

In 12.2% of all patients undergoing total arterial CABG, EC units were transfused perioperatively. Mixed CABG was associated with a significantly higher incidence of EC transfusion (30.1%, *p* < 0.001). In crude comparison, the mixed CABG group showed higher incidence of TC transfusion and FFP transfusion than the total arterial CABG group (*p* = 0.006 and *p* = 0.003, respectively). The quantity of transfused EC units, TC units and FFP units was significantly lower in total arterial CABG (2 vs. 5 EC-units at the 95th percentile, *p* < 0.001; 0 vs. 2 TC-units at the 97th percentile, *p* = 0.007; and 0 vs. 3 FFP-units at the 97th percentile, *p* = 0.003) (Fig. 2).

**Fig. 3:**
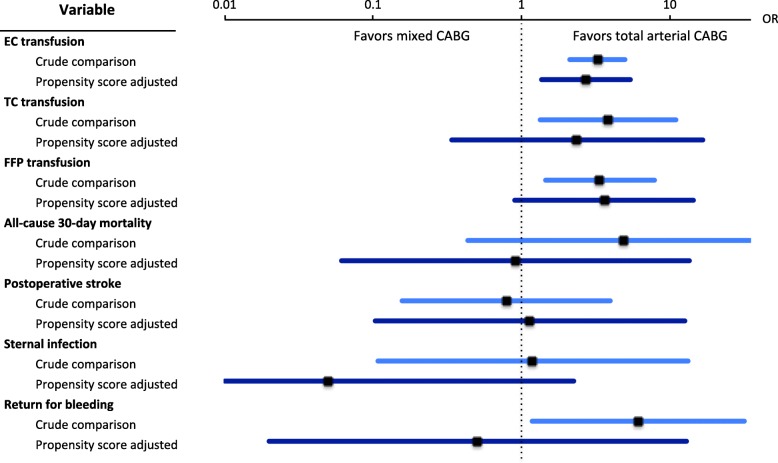
Forest plot indicating odds ratios and 95% CI of blood consumption and early outcomes comparing patients undergoing total arterial CABG (*N* = 419) to mixed CABG (*N* = 176). Results from crude comparison (clear blue) and after propensity score adjustment (dark blue) are depicted in juxtaposition with each other. CI: confidence interval, EC: erythrocyte concentrate, FFP: fresh frozen plasma, TC: thrombocyte concentrate

Propensity score adjusted comparisons are shown in Table 3 and Fig. 3. Propensity score adjustment showed a Hosmer-Lemeshow test *p* value of 0.679. After adjustment, there was a significantly lower incidence of EC transfusion in patients undergoing total arterial CABG compared to mixed CABG (odds ratio [OR] 2.74, 95% confidence interval [CI] 1.38–5.43, *p* = 0.004). However, there was no statistically significant difference in the incidence of TC transfusions (*p* = 0.39) and FFP transfusions (*p* = 0.07), all-cause 30-day mortality (*p* = 0.99), postoperative stroke (*p* = 0.92), sternal infection (*p* = 0.12) and surgical revision of hemorrhage (*p* = 0.68) in the adjusted samples.

**Table 3:** Crude comparison and propensity score adjustment of blood consumption and early outcomes of patients undergoing mixed CABG (*N* = 176) compared to total arterial CABG (*N* = 419)

Variable	Odds ratio	95% CI (lower)	95% CI (upper)	*p* value
EC transfusion				
Crude comparison	3.25	2.10	5.00	< 0.001
Propensity score adjusted	2.74	1.38	5.43	0.004
TC transfusion				
Crude comparison	3.83	1.34	10.93	0.012
Propensity score adjusted	2.36	0.34	16.65	0.39
FFP transfusion				
Crude comparison	3.37	1.45	7.84	0.005
Propensity score adjusted	3.59	0.90	14.34	0.07
All-cause 30-day mortality				
Crude comparison	4.81	0.43	53.33	0.20
Propensity score adjusted	0.92	0.06	13.55	0.99
Postoperative stroke				
Crude comparison	0.79	0.16	3.96	0.78
Propensity score adjusted	1.14	0.10	12.52	0.92
Sternal infection				
Crude comparison	1.19	0.11	13.23	0.89
Propensity score adjusted	0.05	0.01	2.24	0.12
Return for bleeding				
Crude comparison	6.10	1.17	31.73	0.032
Propensity score adjusted	0.50	0.02	12.85	0.68

*EC* erythrocyte concentrate, *FFP* fresh frozen plasma, *TC* thrombocyte concentrate

Examining the effect of single risk factors, lower preoperative hemoglobin levels (*p* < 0.001), older age (*p* < 0.001), female sex (*p* < 0.001) and arterial hypertension (*p* = 0.016) were associated with increased EC transfusion rates, whereas no such association was found in diabetes (*p* = 0.28) and obesity (BMI > 30 kg/cm^2^, *p* = 0.21). Patients receiving four or more distal anastomoses did not show increased EC transfusion rates compared to patients in which three or less distal anastomoses were required (*p* = 0.052). Active medical treatment with Vitamin K antagonists and ADP receptor inhibitors formed risk factors for increased EC transfusion rates (*p* = 0.001 and *p* = 0.018, respectively). No significant association with greater EC transfusion rates was found with Acetylsalicylic acid (*p* = 0.76), Factor Xa inhibitors (*p* = 0.13) and GPIIb/IIIa inhibitors (*p* = 0.81).

## Discussion

This present single-center study compares two different CABG strategies: total arterial CABG using BIMA and mixed CABG using a composition of SIMA and SV conduits, in relation to periprocedural blood loss. The main finding after propensity score adjustment was that patients with multi-vessel coronary artery disease who underwent elective, isolated total arterial CABG using exclusively skeletonized BIMA demonstrated a significantly reduced incidence of EC transfusion.

Our data from baseline profiles indicate a mostly homogenous distribution of cardiovascular risk factors, anticoagulant and antiaggregant therapy, preoperative hemoglobin levels and crossclamp times between the two groups. The number of distal anastomoses was significantly lower in total arterial CABG compared to mixed CABG (3.1 ± 0.9 and 3.9 ± 0.9, respectively). However, complete revascularization was the first priority in our grafting strategy and a hybrid procedure with single percutaneous coronary intervention to the right coronary artery was performed, if the goal of a complete revascularization could not be achieved by BIMA.

After propensity score adjustment, our results demonstrate a 2.7-fold increased incidence of blood transfusions in mixed compared to total arterial CABG. Moreover, a significant reduction of the quantities of transfused EC units was found with the total arterial approach. No difference in the incidence of TC- and FFP-transfusion was found in the propensity-adjusted analysis.

In order to explain these findings, we identified two main mechanisms that lead to blood loss after CABG surgery: First, there are cardiac causes, such as bleeding from cannulation sites, bleeding from proximal or distal anastomoses or bleeding from the bypass conduit itself, e.g. after inadequate clipping of side branches. These causes usually result in substantial hemorrhage and require urgent surgical revision. Second, there are non-cardiac causes, such as bleeding of the sternotomy, bleeding of the IMA-bed or bleeding into the subcutaneous tissue after SV harvesting. Contrary to the cardiac causes, these causes produce a diffuse, gradually progressing blood loss.

In our study population, no significant difference in the incidence of surgical revision of hemorrhage was present between the two groups after propensity score adjustment. We therefore presume that the reduced rates of EC transfusion in total arterial CABG originate from a reduction in non-cardiac causes of blood loss. It has been demonstrated in various studies that BIMA harvesting results in a deteriorated pre- and retrosternal microcirculation [19–21]. After BIMA harvesting, sternal bone perfusion is merely based on the blood flow from intercostal arteries. Consequently, decreased sternal blood flow in total arterial CABG using BIMA may lead to reduced bleeding of the sternotomy. Furthermore, in contrary to every other graft type, no second or extended skin incision is required for IMA graft harvesting. While SV harvesting was always performed using the open technique in this study, we believe that endocopic SV harvesting would not have attenuated the blood consumption in the mixed CABG group. In fact, previous studies comparing endoscopic SV harvesting to open SV harvesting have demonstrated no significant difference in the incidence of red blood cell transfusions [22] or hematoma formation [23]. We are convinced that also the surgeon’s experience and meticulousness is a crucial factor for perioperative bleeding and the consumption of blood products. Since in this present study, all operations were performed by or under supervision of the attending surgeon (A.K.), the bias from different surgeons is minimized.

Contrary to previous findings by Karthik et al. [24], the number of distal anastomoses did not show an independent association with blood transfusions in our study population. Patients receiving four or more distal anastomoses did not show a higher incidence of blood transfusion compared to patients receiving three or less distal anastomoses. Moreover, while Karthik et al. [25] described an increased risk for excessive bleeding with preoperative use of Acetylsalicylic acid, no significant association with greater transfusion rates was found in our population. In accordance to previous studies, where the use of ADP receptor inhibitors in addition to Acetylsalicylic acid has been an independent predictor of EC transfusions and re-operation for bleeding, active medical treatment with ADP receptor inhibitors was as associated with increased EC transfusion rates in our univariable analysis [26, 27].

An argument that is often used against the utilisation of BIMA conduits in total arterial CABG is its association with an increased rate of deep sternal wound infections [19, 27]. However, neither in crude nor in propensity score adjusted analysis did our data show any significant difference in the incidence of deep sternal wound infections between the total arterial and the mixed CABG group.

### Study restrictions

This study is subject to several limitations that demand sensible interpretation of the findings. First, this study is a single-center experience with reduced external validity. Second, with this retrospective study we were unable to show causality. This can only be determined through the design of a randomized controlled trial. Third and final, even though anticoagulation and antiaggregation scheme was closely analyzed, we did not assess specific coagulation factor levels and therefore are unable to identify the impact of secondary hemostasis, clot stability and fibrinolysis on perioperative blood consumption.

## Conclusions

Reduced rates of red blood cell transfusion were found in patients who underwent elective, isolated total arterial CABG for multi-vessel coronary artery disease using exclusively skeletonized BIMA conduits compared to mixed CABG using a combination of SIMA and SV conduits. Our findings suggest that in respect to perioperative blood loss, the use of BIMA in total arterial CABG is safe and can also be applied in patients with a low tolerance of anemia or patients who will not accept allogeneic blood transfusion.

## Data Availability

The datasets generated and/or analysed during the current study are not publicly available due to the individual privacy of the participants, but are available from the corresponding author on reasonable request and with permission of the local research ethics committee.

## Abbreviations

BIMA: Bilateral internal mammary artery
CABG: Coronary artery bypass grafting
EC: Erythrocyte concentrate (packed red blood cells)
FFP: Fresh frozen plasma
IMA: Internal mammary artery
LIMA: Left internal mammary artery
LVEF: Left ventricular ejection fraction
RIMA: Right internal mammary artery
SIMA: Single internal mammary artery
SV: Saphenous vein
TC: Thrombocyte concentrate
